# Neuromuscular or Sensory Electrical Stimulation for Reconditioning Motor Output and Postural Balance in Older Subjects?

**DOI:** 10.3389/fphys.2021.779249

**Published:** 2022-01-12

**Authors:** Thierry Paillard

**Affiliations:** Laboratoire Mouvement, Equilibre, Performance et Santé, EA 4445, Département STAPS, Université de Pau et des Pays de l’Adour, E2S, Tarbes, France

**Keywords:** aging, electrical stimulation, muscle electrical stimulation, somatosensory electrical stimulation, muscle strength, balance, fall, elderly

## Abstract

Percutaneous electrical stimulation is used for reconditioning functional capabilities in older subjects. However, its optimal application depends on the specific physiological needs of the individual. Depending on whether his/her needs are related to motor function or sensory and central functions, the relevant modality of electrical stimulation differs significantly. In fact, there are two main modalities of electrical stimulation, that is, neuromuscular electrical stimulation (NMES) and sensory electrical stimulation (SES). NMES involves high-intensity currents (above the motor threshold) and provokes involuntary visible direct muscle contractions. With chronic application, the induced adaptations occur mainly at the neuromuscular function level and thus enhance muscle strength/power and motor output. SES involves low-intensity currents (below, at or only just above the sensory threshold), does not induce any visible muscle contraction and provides only sensory information. With chronic application, the induced adaptations occur at the level of potentiation and transmission of proprioceptive afferents and thus facilitate sensorimotor activity (movement and balance). Overall, SES is interesting for the improvement/maintenance of sensorimotor capabilities in non-frail older subjects while NMES is relevant to develop muscle strength/power and thus reduce the risk of falls due to a lack of muscle strength/power in frail older subjects.

## Introduction

Advancing age engenders progressive structural and functional alterations of different organs and systems linked to the motor and postural functions. These alterations naturally and chronologically are likely to lead to motor and postural disturbances related, first, to functional capabilities as part of maximal/intense physical activities, second, to basal functional capabilities as part of activities of daily living, and third, to frailty and an increased risk of falling as part of different body displacements and domestic motor actions – activities at home (ANSES, 2016). A sedentary or inactive life accentuates and accelerates these motor and postural alterations. Although regular physical activity and exercise are the best way of preventing, slowing down or limiting these progressive alterations and maintaining the whole functional capabilities (Intiso et al., 2012; Hafström et al., 2016), older subjects are often unable or unwilling to engage in conventional physical activity and exercise or to undertake whole-body physical activity (Paillard, 2018). Hence, in older subjects, in order to limit motor and postural alterations, the optimal (only) solution seems to be the use of artificial techniques, such as the percutaneous peripheral electrical stimulation (Paillard, 2018). This type of stimulation enables the artificial activation of the motor pathway (peripheral and/or central stimulation) and/or the sensory pathway that ensure command, control and execution of movements (Paillard, 2018, 2020, 2021).

**Figure 1:**
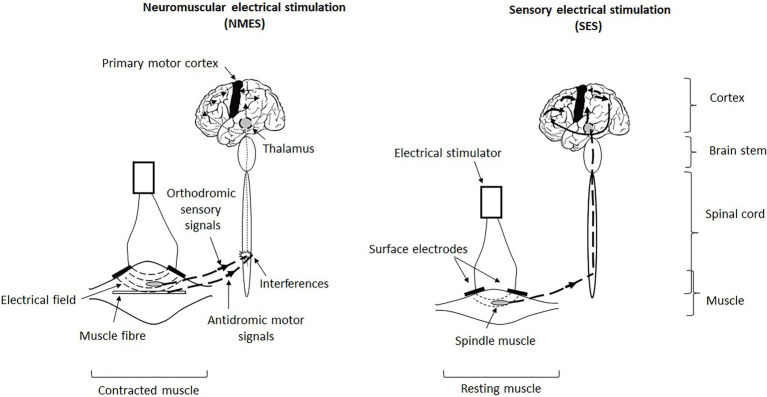
Illustration of the triggering, transmission and central integration of signals generated by neuromuscular electrical stimulation (NMES) and sensory electrical stimulation (SES). NMES involves an excito-motor current, that is, it is strong enough to directly contract the muscle (contracted muscle), whereas SES involves a current below the motor threshold and thus does not induce muscle contraction (resting muscle). In fact, NMES simultaneously activates both sensory and motor neurons and causes a conflict between antidromic motor action potentials and orthodromic sensory action potentials at the spinal level, which interferes with the central integration of the induced afferents (indicated in the diagram of the spinal cord) and thus reduces the ascending sensory volley at the supraspinal level (thin ascending dotted line and thin arrows at cortical level). Hence, NMES generates little exploitable sensory information by the central nervous system to optimise and refine motor and postural skills. In return, SES stimulates only (or almost only) sensory neurons linked to mechanoreceptors which triggers proprioceptive afferents that go up *via* the thalamus to the postcentral and parietal cortices (primary somatosensory cortex and posterior parietal cortex) to precentral cortices (primary motor cortex) in the stimulated hemisphere (thick ascending dotted line and thick arrows at cortical level). Hence, SES generates enhanced sensorimotor activity that is likely to improve motor and postural capabilities.

However, there are two types of percutaneous peripheral electrical stimulation which, through surface electrodes placed over the bellies or motor points of one (or more) superficial skeletal muscle(s), allow either direct activation of the muscle fibres (i.e., excito-motor stimulation that activates not only the motor and sensory nerve fibres but also the muscle fibres directly) or activation of the sensory nerve fibres only (i.e., sensory stimulation that does not activate muscle fibres and motor nerve fibres; Collins, 2007). Stimulation of sensory nerve fibres can also be applied directly to a nerve – for example, femoral or tibial nerve – or to a joint – for example, hip, knee and ankle (Yoshida et al., 2015; Saadat et al., 2017). Excito-motor stimulation is named neuromuscular electrical stimulation (NMES) while sensory stimulation is called sensory (or somatosensory) electrical stimulation (SES). In fact, the type of a stimulation depends on the intensity of the current applied (cf. paragraphs Application modalities and Motor and postural adaptations; Figure 1). Each stimulation induces specific physiological effects on the sensory, central and motor functions related to movement and postural balance with chronic applications (Schröder et al., 2018; Paillard, 2020, 2021). The choice of the stimulation should be based on the specific physiological needs of the older subjects under consideration.

The aim is to propose the type of electrical stimulation best suited to the different physiological profiles of older subjects while specifying the effects induced for each technique and suggesting the underlying mechanistic explanations.

## Physiological Profiles and Specific Reconditioning Needs in Older Subjects

Depending on the physiological profile of older subjects, there are different reconditioning needs for movement and balance. The first physiological profile of advancing age (from a chronological viewpoint) mentioned above (physiological alteration) can be characterised by impaired proprioceptive (myotendinous and articular cues), vestibular (otolithic and semicircular cues) and exteroceptive (visual and skin cues) input and/or integration and/or decreased motor output (Maitre et al., 2013). With this physiological profile, the needs are mainly sensorimotor. Moreover, since the absence of physical activity accentuates all the impairments mentioned above, the functional capabilities are primarily and clearly impeded due to excessive degradation of motor output (Paillard, 2017b; Trajkov et al., 2018). This corresponds to the second physiological profile described above and its needs are primarily based on the development of muscle strength/power (pure motor output) at least in order to partially recondition the basal functional capabilities more easily (Paillard, 2017b). For the third physiological profile corresponding to an increased fall risk, evidence suggests that the needs are the same but are still much more pronounced than those in the second physiological profile related to the absolute needs to develop muscle strength/power (Paillard, 2017b). Whether it is a question of developing sensorimotor function or only motor function (e.g., muscle strength/power), the technique of electrical stimulation constitutes an excellent means for reconditioning older subjects corresponding to the second and third physiological profiles, but it can also be appropriately used with subjects of the first physiological profile, especially when they are sedentary or inactive. However, the type of electrical stimulation required depends on whether the aim is to develop sensorimotor function or only motor function.

## Motor and Postural Adaptations Induced By Electrical Stimulation

### Neuromuscular Electrical Stimulation

#### Application Modalities

NMES is excito-motor which means that it directly activates muscle fibres by bypassing motor neurons (even if they are simultaneously activated as well as sensory neurons). To this end, it must be clearly above the motor threshold, that is, the minimal intensity of stimulation that produces a direct motor response and generates involuntary non-controlled segmental movements. It turns out that the higher the intensity, the greater the extent of the electrical field and the greater the number of recruited muscle fibres – that is, both I and II fibre types (Collins, 2007). In the context of regular or chronic application, it appears that the higher the current intensity, the greater the physiological effects/benefits induced (Paillard, 2018). NMES produces strong muscle contractions and provokes pain through surface electrodes placed over the bellies or motor points of muscle(s) targeted (Lake, 1992; Vanderthommen and Duchateau, 2007). In order to recondition the basal functional capabilities in older (frail) subjects, NMES should be applied specifically to the muscles of their lower limbs with high intensities (maximal tolerable by subject), high frequencies (>30 Hz and rather 50–80 Hz), optimal width pulses (matching to the chronaxy of the stimulated muscle that is, for instance 300–450 μs for quadriceps femoris) short contractions interspersed with long recovery times – for example, 3–10 s/10–30 s on/off – for 10–15 min, 20 min maximum (Paillard, 2018).

#### Motor and Postural Adaptations

Evidence suggests that NMES regularly applied on quadriceps femoris or dorsi/plantarflexor muscles (e.g., tibialis anterior, and soleus) in older subjects (>60 years old) improves lower-limb muscle strength (Caggiano et al., 1994; Paillard et al., 2004; Bezerra et al., 2011; Caulfield et al., 2013; Kern et al., 2014; Mignardot et al., 2015; Von Stengel et al., 2015; Mani et al., 2018; Acaröz Candan et al., 2019; Langeard et al., 2021) and postural balance (Amiridis et al., 2005; Paillard et al., 2005a,b; Nejc et al., 2013; Mignardot et al., 2015; Alptekin et al., 2016; Bondi et al., 2021).

**Figure 2:**
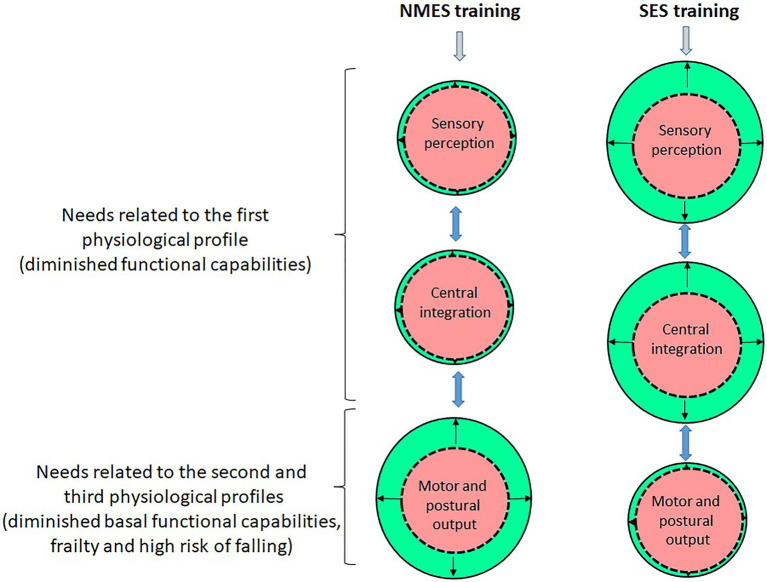
Illustration of the chronic adaptations related to the sensory, central and motor functions induced by NMES and SES (the red/central circles on the figure correspond to the initial condition – that is, pre-training – and the green/peripheral circles correspond to the final condition – that is, post-training; the difference between the red/central and green/peripheral circles corresponds to the amplitude of adaptations induced by NMES or SES). NMES mainly stimulates the neuromuscular system and especially induces muscle structural and nerve functional adaptations that enhance muscle strength/power as well as motor output related to movement and postural balance. Since the induced ascending sensory volleys are reduced due to interference with antidromic motor signals, NMES generate little adaptation at the sensory and central levels. SES alters the sensibility threshold of mechanoreceptors in the long term that optimises the potentiation and transmission of proprioceptive afferents and thus enhances the sensory contribution which facilitates sensorimotor activity and motor cortex excitability related to movement and postural balance. In older subjects in the first physiological profile, their need to improve their diminished functional capabilities is more related to the implementation of SES, while in frail older subjects in the second and third physiological profiles, their need to reduce/reverse their frailty and risk of falling is more related to the implementation of NMES.

In this context, the functional improvements of motor and postural functions in older subjects are mainly linked to enhancements of motor output through muscle structural (mass) and functional (neural networks) adaptations – Figure 2 (Paillard, 2018). The naturally irreversible atrophy of lower-limb muscle in inactive or sedentary individuals can be reversed with the chronic application of NMES (Boncompagni et al., 2007; Carraro et al., 2015). NMES stimulates not only anabolic pathways (e.g., secretion of insulin-like growth factor-1), but also negatively modulates muscle catabolism, which increases protein synthesis and reduces protein degradation and activates satellite cells in aged individuals (Barberi et al., 2015; Mancinelli et al., 2019). Hence, NMES induces an increase in the size of muscle fibres especially in the type II muscle fibres which are particularly affected by the effects of advancing age (Zampieri et al., 2015; Mancinelli et al., 2019). From a functional viewpoint, NMES results in greater contribution of muscles regularly stimulated (i.e., electromyographic activity of tibialis anterior and medial gastrocnemius) in the postural regulation (Amiridis et al., 2005). It also entails increased musculotendinous stiffness of the muscles regularly stimulated (Mignardot et al., 2015).

However, even if one cannot ignore that NMES applied on selected muscle groups engenders sensory information likely to be used as part of the command and control of voluntary movement – for example, during walking – Paillard (2020) inferred that it generates little sensory information likely to be exploited by the central nervous system to optimise and refine perceptual and motor skills as part of the postural balance regulation (Figure 2). In fact, NMES simultaneously activates both sensory and motor neurons and would provoke conflict between the antidromic motor action potentials and the orthodromic sensory action potentials at the spinal level (Bergquist et al., 2011), thus causing interference in the central integration of induced afferents (although the presence of central adaptations cannot be totally excluded).

In order to improve the functional capabilities, NMES would be particularly interesting since it is likely to enhance motor output especially muscle strength/power in older frail subjects with a high risk of falling precisely due to a lack of muscle strength/power.

### Sensory Electrical Stimulation

#### Application Modalities

SES is not excito-motor (it cannot directly activate the muscle fibres) and only (or almost only) activates the sensory pathways (this is the principle of this stimulation). To this end, it must be well below the motor threshold and close to the sensory threshold, that is, the minimum intensity of stimulation that can be perceived by individual. Indeed, the current intensity must be around the sensory threshold without exceeding it or only slightly, since a non-excito-motor current (low intensity) is already likely to lead to recruitment of type I muscle fibres from reflex pathways – mediated at spinal level, that is, homolateral monosynaptic connections of the Ia fibres with the α-motoneurons generate depolarisation of the latter and induce the contraction of the muscle fibres they innervate (Zeronian et al., 2021). SES is painless (although there are possible sensations) and applied to a peripheral nerve, belly muscles (motor points) and/or joints at current intensities below, at or slightly above the sensory threshold (Schröder et al., 2018; Paillard, 2021). In order to recondition the motor and postural functions in older subjects, SES should be applied with large pulses (e.g., 1 ms) and high frequencies (80–100 Hz) to facilitate potentiation and central integration of emitted signals (Collins, 2007; Bergquist et al., 2011) for several tens of minutes in a uninterrupted way.

#### Motor and Postural Adaptations

Evidence suggests that SES regularly applied to lower-limb muscle (motor points), peripheral nerve or joints in older subjects (>60 years old) improves sensorimotor function (Park et al., 2014; Ng et al., 2016; Paillard, 2021). However, since SES generates no muscle deformation or contraction, it thus cannot stimulate synthesis of contractile proteins responsible for muscle structural adaptations (Coffey and Hawley, 2007). The adaptations induced cannot occur at the level of the motor output of muscle. In fact, SES generates sensory cues that are detected by sensory sensors which transmit signals throughout sensory pathways to cortical areas (Figure 1). With chronic application, SES induces adaptations at different stages (sites) throughout sensory pathways – sensory sensors and spinal and supraspinal structures (Figure 2).

At the peripheral level (i.e., sensory sensors), SES would modify the sensitivity threshold of mechanoreceptors that result from altering the ion permeability of their membrane (Gravelle et al., 2002; Ross et al., 2007). This sensory adaptation would optimise the potentiation and transmission of proprioceptive afferents and thus enhance the sensory contribution in the motor and postural regulation (Ross et al., 2007; Severini and Delahunt, 2018; Paillard, 2021). At the spinal level, SES is likely to engender a reduction or increase in the amplitude of the induced H-reflex (Goulet et al., 1997; Hardy et al., 2002, respectively) that, in both cases, can improve motor and postural abilities (Paillard, 2017a). The reduction of the Ia-afferent excitation to the α-motoneuron pool can attenuate destabilising joint movements while the enhancement of the activation of the α-motoneuron pool can facilitate instantaneous segmental reactions to the demands of the motor or postural task (Paillard, 2017a). At the cortical level, SES is likely to induce durable changes in motor cortex excitability that can be assimilated to enhance sensorimotor activity and connectivity (Kaelin-Lang et al., 2002; Schröder et al., 2018; Insausti-Delgado et al., 2021). Enhanced sensorimotor activity could result from favourable impact of SES over postcentral and parietal cortices (primary somatosensory cortex and posterior parietal cortex) to precentral cortices (primary motor cortex) in the stimulated hemisphere (Paillard, 2021).

SES would be of particular interest as it is likely to improve sensorimotor output in older subjects who are not at high risk of falling due to lack of muscle strength/power or very weak motor output.

## Which Electrical Stimulation for Which Physiological Profile in Older Subjects?

On the basis of an older subject’s needs, belonging to the first physiological profile, SES would turn out to be particularly interesting since it especially facilitates the sensorimotor reconditioning and thus optimises functional capabilities (Figure 2). For older subjects in the second physiological profile who exhibit diminished basal functional capabilities, NMES would turn out to be relevant since it can initiate/trigger structural and functional muscle adaptations that can reverse the process of reduction of functional capabilities in activities of daily living (Figure 2). Regarding older subjects in the third physiological profile who are at high risk of falling due to extreme frailty and a notable lack of muscle strength/power in the lower-limb, NMES would reverse the process of frailty and muscle involution, thus reducing the risk of falling.

## Conclusion

In older subjects, as long as their basal functional capabilities are not clearly limited/reduced as part of activities of daily living and their risk of falling is low, SES is particularly useful for maintaining or even improving their capabilities to command and control movement and postural balance. In turn, in frail older subjects with diminished basal functional capabilities and at high risk of falling, NMES can potentially boost their neuromuscular system in order to recondition the lower-limb muscle strength/power thus limiting their risk of falling.

## Author Contributions

The author confirms being the sole contributor of this work and has approved it for publication.

## Conflict of Interest

The author declares that the research was conducted in the absence of any commercial or financial relationships that could be construed as a potential conflict of interest.

## Publisher’s Note

All claims expressed in this article are solely those of the authors and do not necessarily represent those of their affiliated organizations, or those of the publisher, the editors and the reviewers. Any product that may be evaluated in this article, or claim that may be made by its manufacturer, is not guaranteed or endorsed by the publisher.
